# Impact of Robotic-Assisted Thoracic Surgery on the Completion of Adjuvant Chemotherapy Following Lung Cancer Resection

**DOI:** 10.7759/cureus.30364

**Published:** 2022-10-16

**Authors:** Khaled Abdelghany, Lary A Robinson, Tawee Tanvetyanon

**Affiliations:** 1 Morsani College of Medicine, University of South Florida, Tampa, USA; 2 Thoracic Oncology Department, H. Lee Moffitt Cancer Center, Tampa, USA

**Keywords:** retrospective studies, propensity score matching (psm), robotic surgery, adjuvant chemotherapy, lung cancer

## Abstract

Background

Adjuvant chemotherapy can further improve treatment outcomes following the resection of non-small cell lung cancer (NSCLC). However, in practice, some patients are unable to tolerate all prescribed chemotherapy. One of the factors which may implicate adjuvant chemotherapy completion is a surgical technique. We investigated the impact of robotic-assisted thoracic surgery (RATS), a form of minimally invasive surgery, on chemotherapy completion.

Methods

We conducted a retrospective study of NSCLC patients who underwent adjuvant platinum-based chemotherapy at our institution during 2010-2020. The primary outcome of interest was chemotherapy completion, defined as receiving all 4 cycles of chemotherapy. We also performed an exploratory analysis to identify factors associated with chemotherapy completion.

Results

Analyses included 165 patients: 95 patients underwent traditional thoracotomy, and 70 patients underwent RATS. Baseline characteristics were comparable except for smaller tumor size and lower stage in the RATS group. Median operative time was longer in the RATS group than in the thoracotomy group: 198 vs. 139 minutes, *p*<0.001. Chemotherapy completion rates were not significantly different between groups: 74.3% vs. 75.8%, *p*=0.83, respectively. In addition, no significant difference was found in the incidences of postoperative complications between groups. In a propensity score matched analysis, there was also no difference in the chemotherapy completion rates between groups. Multivariable logistic regression analysis indicated that independent factors predicting completion of adjuvant chemotherapy were body mass index, postoperative complications, year of treatment, and T-stage.

Conclusion

In this large cohort of NSCLC patients who received adjuvant chemotherapy, no association was found between surgical technique and adjuvant chemotherapy completion.

## Introduction

Robotic-assisted thoracic surgery (RATS) has been increasingly adopted in the United States [1]. RATS is a newer form of video-assisted thoracic surgery (VATS), a minimally invasive surgical approach in which intrathoracic organs are accessed through several small openings in the chest, rather than an incision [2]. During RATS, the surgeon’s hands remain outside the thoracic cavity throughout, manipulating the robotic arms. Some studies have found that minimally invasive surgical techniques are associated with lower surgical complications, shorter hospital stays, and better adjuvant chemotherapy delivery than traditional thoracotomy [3-5].

The delivery of adjuvant chemotherapy is clinically relevant for non-small cell lung cancer (NSCLC) because adjuvant chemotherapy can improve the cure rate among those with pathological stage IIA or greater [6]. Meta-analyses of large phase-3 clinical trials have shown that adjuvant chemotherapy reduces the absolute mortality risk by 5% [7,8]. In these studies, adjuvant chemotherapy is administered for 4 treatment cycles. Nevertheless, in practice, some patients will not be able to tolerate all prescribed doses. Inability to complete planned chemotherapy dosages may adversely affect its efficacy and factors which can facilitate the completion of adjuvant chemotherapy are of clinical interest.

Because minimally invasive surgery has been associated with reduced surgical complications, we hypothesize that it may also facilitate the completion of adjuvant chemotherapy. The most available literature on minimally invasive thoracic surgery is based on VATS, rather than RATS. Because RATS is a newer form of VATS, it remains unknown whether RATS has any impact on adjuvant chemotherapy completion. Our institution is one of the institutions offering RATS to a large number of patients over the past decade. In this study, therefore, we compared RATS and traditional thoracotomy with the outcome of interest being adjuvant chemotherapy completion. To account for potential differences in the patient and disease characteristics at baseline, regression modeling and propensity-adjusted methods were employed.

## Materials and methods

Patient cohort

After receiving approval from the Scientific Review Committee and the University of Florida Institutional Review Board -MCC20922 IRB study 001747, we retrospectively reviewed the electronic medical records of patients who underwent lung cancer resection at Moffitt Cancer Center between 2010 and 2020. Inclusion criteria were those patients who underwent R0 or R1 resection and received at least 1 cycle of adjuvant chemotherapy at this institution. Adjuvant chemotherapy was defined as cisplatin or carboplatin-based doublet chemotherapy. Exclusion criteria were those treated with neoadjuvant chemotherapy, had known metastatic disease prior to chemotherapy, received concurrent radiotherapy, or had surgery with VATS technique without RATS.

Medical record review

Medical records of eligible patients were reviewed for patient-, cancer- and treatment-related characteristics. The primary outcome of interest was adjuvant chemotherapy completion defined as the receipt of all 4 cycles of chemotherapy. The secondary outcomes were the length of hospital stay, surgical complications, and serious chemotherapy-related complications. Dose reduction or incomplete chemotherapy scheduled was still counted toward the chemotherapy cycle as long as carboplatin or cisplatin was administered. Performance status was assessed according to the Eastern cooperative oncology group scale [9], documented at an outpatient visit prior to the first adjuvant chemotherapy cycle. Comorbidity burden score was calculated by the Charlson comorbidity index [10]. Chemotherapy-related toxicity was graded according to common terminology for adverse event version 4.0 [11]. Serious toxicities were those necessitating hospitalization or were grade 3 or higher. Surgical complications were classified according to the Clavien-Dindo scale [12]. Pathological staging was based on the International Association for the Study of Lung Cancer, version 8 [13].

Treatment procedure

A thoracotomy consists of a 10-12 cm posterolateral rib-spreading incision and direct insertion of instruments and at times the surgeon’s hand into thoracic cavity for the surgical procedures. The technique for RATS has been previously detailed elsewhere [14,15]. Briefly, the system consisted of a 4-cm camera port plus an assistant’s access port and two 1-cm instrument ports. From September 2010 through December 2011, the da Vinci S robotic surgical system was used, followed by the da Vinci Si system from January 2012 to March 2017, and the da Vinci Xi system (Intuitive Surgical Corporation, Sunnyvale, CA) from April 2017 to 2020. Adjuvant chemotherapy regimens consisted of carboplatin or cisplatin plus pemetrexed, docetaxel, vinorelbine, or gemcitabine. In addition, during the study period, bevacizumab was available for some patients through clinical trials. At our institution, adjuvant radiotherapy, when indicated, was given sequentially after adjuvant chemotherapy.

Statistical analysis

Descriptive statistics including mean, median and range for scale variables as well as count and proportion for categorical variables were performed as appropriate. Non-parametric tests were used to compare scale variables and Chi-square, or exact tests were used to compare categorical variables. We performed a propensity score matching analysis to address the imbalance in some of the baseline characteristics between the thoracotomy and robotic groups. Propensity score represented the propensity to have received robotic surgery and was calculated by incorporating stage group, performance status, extent of surgery, and surgery year as predictors, using a caliper matching of 0.1 without replacement. Absolute standardized differences were calculated to assess the balance between groups. Univariable and multivariable logistic regression analyses were performed to examine factors potentially associated with adjuvant chemotherapy completion. All p-values were two-tailed, and significance level was set at <0.05. Analyses and graphics were performed on SPSS, version 24 (IBM corp, Armonk, NY).

## Results

Patient and treatment characteristics

During the study period, 180 patients were initially identified by our search strategy. Of these, 15 patients were excluded due to developing metastatic disease before chemotherapy (n=8), concurrent thoracic radiotherapy (n=3), patient relocation (n=2), small-cell histology (n=1) and neoadjuvant chemotherapy (n=1), leaving 165 patients included in the analyses. Of these, 95 patients received thoracotomy while 70 patients received RATS (Table 1). 

**Table 1:** Baseline patient and treatment characteristics. BMI, body mass index; FEV-1, forced expiratory volume at 1 second; DLCO, diffusing capacity of carbon monoxide; Hb, hemoglobin concentration, Cr, serum creatinine; ASA, American Society of Anesthesiologists physical classification; ECOG, Eastern Cooperative Oncology Group performance status; OR, operating room; SD, standard deviation.

Baseline characteristics	Open N=95 (%)	Robotic N=70 (%)	Total N=165 (%)	p-value
Patient-related:				
Median age, range	66.2, 42.5-81.3	67.7, 48.1-87.0	66.9, 43.6-87.0	0.14
Median BMI, range	27.5, 18.2-51.8	26.5, 17.6-40.4	27.2, 17.6-51.8	0.62
Median FEV-1 liter, range	2.3, 1.2-3.8	2.1, 1.2-3.1	2.2, 1.2-3.7	0.008
Median % predicted FEV-1, range	83.0, 43.0-132.0	81.0, 46.0-113.0	82.0, 43.0-132.0	0.29
Median % DLCO, range	75.0, 42.0-114.0	70.5, 42.0-112.0	73.5, 42.0-114.0	0.12
Median baseline Hb g/dl, range	12.8, 9.8-15.9	13.3, 10.5-15.9	13.2, 9.8-15.9	0.19
Median baseline Cr mg/dl, range	0.8, 0.5-1.4	0.8, 0.4-1.4	0.8, 0.4-1.4	1.00
ASA class at surgery:				
-2	48 (51.1)	36 (52.2)	84 (51.5)	0.48
-3	44 (46.8)	33 (47.8)	77 (47.2)	
-4	2 (2.1)	0 (0)	0 (0)	
ECOG at chemotherapy:				
-0	34 (35.8)	36 (51.4)	70 (42.4)	0.04
-1	61 (64.2)	34 (48.6)	95 (57.6)	
Race				
-White	90 (94.7)	64 (91.4)	154 (93.3)	0.45
-Others	5 (5.3)	6 (8.6)	11 (6.7)	
Sex:				
-Male	46 (48.4)	29 (41.4)	75 (45.5)	0.37
-Female	49 (51.6)	41 (58.6)	90 (54.5)	
Median comorbidity index, range				
Cancer-related:				
-adenocarcinoma	58 (61.1)	43 (61.4)	101 (61.2)	0.54
-squamous cell carcinoma	30 (31.6)	22 (31.4)	52 (31.5)	
-others	7 (7.3)	5 (7.2)	12 (7.3)	
Median tumor size cm, range	4.8, 1.0-12.5	3.8, 0.8-10.0	4.4, 0.8-12.5	0.002
Nodal stage:				
-N0	35 (36.8)	26 (37.1)	61 (37.0)	0.39
-N1	42 (44.2)	36 (51.4)	78 (47.3)	
-N2	18 (18.9)	8 (11.4)	26 (15.8)	
Tumor stage:				
-1a, 1b, 1c	25 (26.3)	22 (31.4)	47 (28.5)	0.19
-2a, 2b	28 (29.5)	26 (37.1)	54 (32.7)	
-3	25 (26.3)	17 (24.3)	42 (25.5)	
-4	17 (17.9)	5 (7.1)	22 (13.3)	
Stage group:				
-Ib	1 (1.1)	1 (1.4)	2 (1.2)	0.002
-IIA, IIB	48 (50.5)	54 (77.1)	102 (61.8)	
-IIIA, IIIB	46 (48.4)	15 (21.4)	61 (37.0)	
Treatment-related:				
Year of surgery:				
-2009-2014	65 (68.4)	27 (38.6)	92 (55.8)	<0.001
-2015-2019	30 (31.6)	43 (61.4)	73 (44.2)	
Type of surgery:				
-bi-lobectomy	4 (4.2)	3 (4.3)	7 (4.2)	<0.001
-lobectomy	68 (71.6)	56 (80.0)	124 (75.2)	
-pneumonectomy	19 (20.0)	0 (0)	19 (11.5)	
-segmentectomy	2 (2.1)	3 (4.3)	5 (3.0)	
-wedge resection	2 (2.1)	8 (11.4)	10 (6.1)	
Positive margins	2 (2.1)	5 (7.1)	7 (4.2)	0.14
Laterality of surgery:				
-Left	41 (43.2)	25 (35.7)	66 (40.0)	0.41
-Right	54 (56.8)	45 (64.3)	99 (60.0)	
Primary anatomic lobe:				
-lower	38 (40.0)	29 (41.4)	67 (40.6)	0.85
-middle	6 (6.3)	3 (4.3)	9 (5.5)	
-upper	51 (53.7)	38 (54.3)	89 (53.9)	
Cisplatin-based regimen	73 (76.8)	55 (78.6)	128 (77.6)	0.78
Doublet chemotherapy:				
-pemetrexed	60 (63.2)	40 (57.1)	100 (60.6)	0.86
-gemcitabine	22 (23.2)	17 (24.3)	39 (23.6)	
-others	13 (13.6)	13 (18.6)	26 (15.8)	

**Figure 1:**
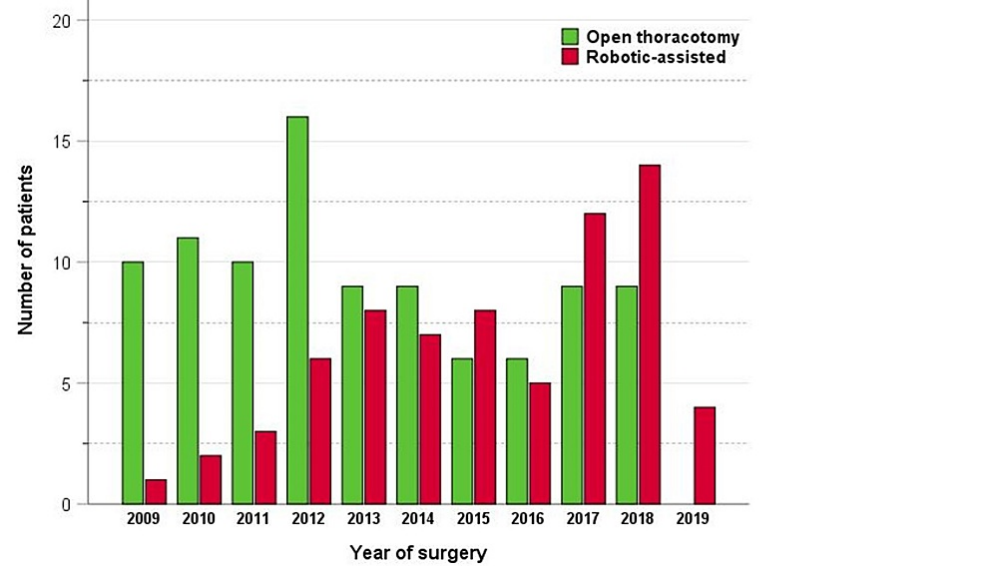
Number of patients by year of surgery.

The median age was 66.9 years. Baseline patient-related characteristics were comparable between groups except for performance status, with a greater proportion of asymptomatic patients in the RATS group. Baseline tumor-related characteristics were similar for histology and nodal stage. However, tumor size was smaller, and the stage was lower in the RATS group. Stage III patients comprised 21.4% in the RATS group, compared with 48.4% in the thoracotomy group, p=0.002. Four patients had stage IIIB: two T3N2 and two T4N2. All nodal involvement was microscopic and T3 or T4 was due to a separate tumor in the same or different, ipsilateral lobe. Baseline treatment characteristics were comparable in laterality, primary anatomic lobe, and adjuvant chemotherapy regimen. The resection type was a lobectomy in 71.6% of the thoracotomy group and 80.0% of the RATS group. Sub-lobar resection was performed more frequently among the RATS group and there was no pneumonectomy. It was notable that RATS was increasingly performed in recent years. During 2009-2014, RATS comprised only 38.6% of the cases; however, during 2015-2019, this increased to 61.4%, p<0.001 (Figure 1).

Clinical outcomes

Operating time was longer in the RATS group compared to the thoracotomy group: median 198 minutes versus 139 minutes, respectively, p<0.001 (Table 2).

**Table 2:** Clinical outcomes. OR, operative room; SD, standard deviation.

Outcomes of interest	Open N=95 (%)	Robotic N=70 (%)	p-value
Surgical outcomes:			
Median OR time minutes, range	139, 78-409	198, 68-386	<0.001
Surgical blood loss ml, range	150, 15-1600	100, 5-600	0.09
Median length of stay days, range	4, 2-15	3, 2-16	0.02
Chemotherapy outcomes			
Median days from surgery to chemotherapy, range	59, 19-105	54, 27-103	0.08
Mean chemo cycle, SD	3.6, 0.8	3.5, 0.9	0.72
Received only one cycle	4 (4.2)	5 (7.1)	0.49
Received all four cycles	72 (75.8)	52 (74.3)	0.83
Any grade-3 toxicity	16 (16.8)	14 (20.0)	0.60
Hospitalization due to toxicity	11 (11.6)	10 (14.3)	0.61

The median length of hospital stay was shorter in the RATS group: 3 days versus 4 days, p=0.02. No significant difference was observed in the incidence of surgical complications between groups (Table 3).

**Table 3:** Surgical complications.

Type of complications	Open N=95 (%)	Robotic N=70 (%)	Total N=165 (%)	p-value
Bleeding:				
Index hospital transfusion	12 (12.6)	7 (10.0)	19 (11.5)	0.60
Intraoperative bleeding	3 (3.2)	2 (2.9)	5 (3.0)	1.00
Postoperative bleeding	3 (3.2)	1 (1.4)	4 (2.4)	0.64
Respiratory:				
Acute respiratory failure	4 (4.2)	1 (1.4)	5 (3.0)	0.39
Air leak	9 (9.5)	13 (18.6)	22 (13.3)	0.09
Subcutaneous emphysema	2 (1.2)	1 (1.6)	3 (1.8)	1.00
Empyema	0 (0)	1 (1.4)	1 (0.6)	0.42
Pneumonia	8 (8.4)	3 (4.3)	11 (6.7)	0.29
Cardiovascular:				
Atrial arrhythmia	15 (15.8)	12 (17.1)	27 (16.4)	0.82
Deep venous thrombosis	1 (1.1)	2 (2.9)	3 (1.8)	0.58
Hypotension	1 (1.1)	0 (0)	1 (0.6)	1.00
Others:				
Chylothorax	0 (0)	1 (1.4)	1 (0.6)	0.42
Urinary tract infection	1 (0.6)	2 (2.9)	3 (1.8)	0.58
Hoarseness or dysphagia	4 (4.2)	3 (4.3)	7 (4.2)	1.00
Overview:				
Repair or revision within 30 days	3 (3.2)	3 (4.3)	6 (3.6)	0.69
Readmission within 30 days	3 (3.2)	3 (4.3)	6 (3.6)	0.69
Clavien-Dindo grade:				
Grade 0	48 (50.5)	38 (54.3)	86 (52.1)	0.63
Grade I	7 (7.4)	5 (7.1)	12 (7.3)	0.96
Grade II	29 (30.5)	17 (24.3)	48 (27.9)	0.38
Grade III	4 (4.2)	5 (7.1)	9 (5.5)	0.49
Grade IIIa	1 (1.1)	1 (1.4)	2 (1.2)	1.00
Grade IIIb	6 (6.3)	3 (4.3)	9 (5.5)	0.73
Grade IV	0 (0)	1 (1.4)	1 (0.6)	0.42

Overall, surgical complications of Clavien-Dindo grade ≥3 occurred in 12.8%, comparable in both groups. No operative mortality occurred.

Completion of chemotherapy was achieved in 124 patients (75.2%). There was no difference in the proportion of patients completing chemotherapy between groups: 75.8% in the thoracotomy group versus 74.3% in the RATS group, p=0.83. The mean number of chemotherapy cycles was 3.6 cycles in the thoracotomy group, compared with 3.5 cycles in the RATS group. Furthermore, no significant difference was observed between groups in the incidence of hospitalization related to adjuvant chemotherapy as well as serious chemotherapy-related toxicity of grade-3 or higher. There was a non-significant trend toward a shorter time to initiation of adjuvant chemotherapy in the RATS group.

Of the 41 patients who did not receive all 4 cycles of chemotherapy, the reasons for discontinuation were side effects (n=33), refusal to continue (n=5), progressive disease (n=2), and death (n=1). Side effects resulting in premature discontinuation included fatigue, stroke, anemia, skin rash, thrombophlebitis, neutropenic sepsis, myocardial infarction, pulmonary embolism, and diabetic ketoacidosis. One patient died of an unknown cause after receiving one cycle of chemotherapy. Of note, cisplatin was initially prescribed in 128 patients (77.6%). However, in 13 patients, cisplatin was subsequently changed to carboplatin due to tinnitus (n=6), nausea or vomiting (n=5), and renal insufficiency (n=2).

Propensity-matched analysis

Following our matching algorithm, 90 patients with closely matched propensity scores were identified: 45 in the thoracotomy group and 45 in the robotic group (Table 4).

**Table 4:** Propensity-matched analyses. BMI, body mass index; FEV-1, forced expiratory volume at 1 second; DLCO, diffusing capacity of carbon monoxide; Hb, hemoglobin concentration; ECOG, Eastern Cooperative Oncology Group performance status; OR, operating room; SD, standard deviation.

Variables	Open N=45 (%)	Robotic N=45 (%)	Total N=90 (%)	p-value
Baseline characteristics:				
Sex:				
-Male	20 (44.4)	20 (44.4)	40 (44.4)	1.00
-Female	25 (55.6)	25 (55.6)	50 (55.6)	
Median age, range	66.9, 44.7-80.9	67.5, 48.1-87.0	67.3, 44.7-87.0	0.76
Median BMI, range	27.6, 18.2-51.8	25.9, 17.6-39.8	27.0, 17.6-51.8	0.22
Median FEV-1 liter, range	2.3, 1.3-3.7	2.1, 1.2-3.1	2.2, 1.2-3.7	0.09
Mean Charlson index, SD	0.7, 0.7	0.5, 0.8	0.6, 0.7	0.41
ECOG at chemotherapy:				
-0	19 (42.2)	25 (55.6)	44 (48.9)	0.27
-1	26 (57.8)	20 (44.4)	46 (51.1)	
Median tumor size cm, range	3.2, 1.2-12.5	4.0, 0.8-10.0	3.6, 0.8-12.5	0.62
Nodal stage:				
-N0	16 (35.6)	15 (33.3)	31 (34.4)	0.77
-N1	21 (46.7)	24 (53.3)	45 (50.0)	
-N2	8 (17.8)	6 (13.3)	14 (15.6)	
Tumor stage:				
-1a, 1b, 1c	19 (42.2)	12 (26.7)	31 (34.4)	0.29
-2a, 2b	17 (37.8)	17 (37.8)	34 (37.8)	
-3	7 (15.6)	11 (24.4)	18 (20.0)	
-4	2 (4.4)	5 (11.1)	7 (7.8)	
Stage group:				
-IB	1 (2.2)	1 (2.2)	2 (2.2)	0.77
-IIA, IIB	34 (75.6)	31 (68.9)	65 (72.2)	
-IIIA, IIIB	10 (22.2)	13 (28.9)	23 (25.6)	
Treatment characteristics:				
Year of surgery 2015-2019	14 (31.1)	21 (46.7)	35 (38.9)	0.13
Type of surgery:				
-sublobar resection	4 (8.9)	2 (4.4)	6 (6.7)	0.39
-lobectomy, bilobectomy	41 (91.1)	43 (95.6)	64 (93.3)	
-pneumonectomy	0 (0)	0 (0)	0 (0)	
Cisplatin-based regimen	34 (75.6)	34 (75.6)	68 (75.6)	1.00
Surgical outcomes:				
Median surgical blood loss ml, range	100, 15-700	100, 20-550	100, 15-700	0.67
Median OR time minutes, range	134, 78-306	203, 68-386	167, 68-386	<0.001
Median length of stay days, range	4, 3-15	3, 2-13	4, 2-15	0.10
Repair or revision within 30 days	3 (6.7)	1 (2.2)	4 (4.4)	0.63
Clavien-Dindo grade 3 or worse	7 (15.6)	5 (11.1)	12 (13.3)	0.53
Clavien-Dindo grade 0	22 (48.9)	25 (55.6)	47 (52.2)	0.53
Atrial arrhythmia	8 (17.8)	8 (17.8)	16 (17.8)	1.00
Chemotherapy outcomes:				
Median days from surgery to chemotherapy, range	62, 19-105	56, 27-103	58, 19-105	0.11
Mean chemotherapy cycle, SD	3.5, 0.9	3.5, 0.9	3.5, 0.9	0.65
Received all four cycles	32 (71.1)	31 (68.9)	63 (70.0)	0.83
Any grade ≥3 toxicity	10 (22.2)	9 (20.0)	19 (21.1)	0.82
Hospitalization due to toxicity	4 (8.9)	6 (13.3)	10 (11.1)	0.48

Baseline characteristics in this patient cohort were well balanced. Pneumonectomy patients were not selected for this analysis. The tumor size and tumor stage were comparable between groups as well as the year of surgery. The absolute standardized difference in the proportion of patients undergoing surgery after 2015 onward was 0.63 in the original cohort. However, this decreased to 0.32 in this cohort.

Again, no difference was found in the proportion of patients completing adjuvant chemotherapy between the thoracotomy and robotic groups. There was no difference observed in the serious toxicity from chemotherapy. Furthermore, no difference in surgical complications or surgical blood loss was observed. The length of hospital stay was no longer significantly different between groups.

Factors influencing adjuvant chemotherapy completion

We performed an exploratory analysis to identify factors influencing the completion of chemotherapy (Table 5).

**Table 5:** Predictors of adjuvant chemotherapy completion. *Variables included in the multivariable analysis BMI, body mass index; FEV-1, forced expiratory volume at 1 second; ECOG, Eastern Cooperative Oncology Group performance status; NS, not significant; NA, not selected in the final model.

Factors	Univariable analysis		Multivariable analysis	
	Odds ratio (95% CI)	p-value	Odds ratio (95% CI)	p-value
Patient-related:				
Age ≥65 years	1.36 (0.67-2.77)	0.39	NA	NS
Male sex	0.95 (0.47-1.94)	0.89	NA	NS
BMI ≥30	0.41 (0.19-0.87)	0.02*	0.39 (0.17-0.86)	0.020
ECOG ≥1	0.63 (0.30-1.31)	0.22*	NA	NS
Charlson index	0.98 (0.63-1.52)	0.93	NA	NS
FEV1 <1.5 liter	0.64 (0.18-2.24)	0.48	NA	NS
Tumor-related:				
T stage ≥2	0.35 (0.14-0.89)	0.03*	0.26 (0.09-0.74)	0.011
N stage ≥1	1.12 (0.54-2.32)	0.75	NA	NS
Stage group ≥III	1.02 (0.49-2.13)	0.95	NA	NS
Adenocarcinoma	0.86 (0.42-1.77)	0.69	NA	NS
Treatment-related:				
Pneumonectomy	0.92 (0.31-2.72)	0.88	NA	NS
Year of treatment	1.13 (0.99-1.28)	0.05*	1.15 (1.01-1.32)	0.037
Robotic surgery	0.92 (0.45-1.88)	0.83	NA	NS
Left lung surgery	1.86 (0.87-3.99)	0.11*	NA	NS
Cisplatin	0.96 (0.41-2.26)	0.93	NA	NS
Hospital stay ≥7 days	0.53 (0.20-1.36)	0.19*	NA	NS
Clavien-Dindo grade ≥3	0.48 (0.18-1.27)	0.14*	0.26 (0.09-0.77)	0.014

We first performed a univariable analysis to examine the relationship between each clinical factor and chemotherapy completion. As previously described, no significant association was found between surgical technique (thoracotomy versus robotic) and chemotherapy completion: Odds ratio (OR) 0.92 (95% CI: 0.45-1.88). In the univariable analysis, factors including BMI, ECOG performance status, T stage, year of treatment, left lung surgery, prolonged hospitalization, and surgical complication were identified as significant or approaching statistical significance and these variables were examined in the multivariable analysis. In the multivariable analysis, only four independent factors were identified: the more recent year of treatment predicted increased chemotherapy completion, but the higher BMI, T-stage >1, and Clavien-Dindo grade ≥3 predicted decreased chemotherapy completion.

## Discussion

In this study, we compared RATS with thoracotomy with the outcomes of interest being adjuvant chemotherapy completion, hospital stay, and chemotherapy-related toxicity. RATS was used more frequently than thoracotomy among patients with smaller tumor sizes and lower stages. In an unadjusted analysis, RATS was associated with a shorter hospital stay than thoracotomy, but not in other outcomes. However, in the propensity score-adjusted analysis, no significant differences between the two groups were observed in any outcomes of interest at all. In all analytic models, operative time for RATS is significantly longer than for thoracotomy. Factors influencing chemotherapy completion were the year of treatment, BMI, T-stage, and presence of surgical complications.

The finding that operative time for RATS is longer than thoracotomy is in line with previous literature [16,17]. Since the RATS technique involves the use of additional instruments which require additional time for set-up and the maneuvering of robotic arms can be time-consuming. As such, this finding is expected. However, the finding that RATS was not associated with a reduced length of hospital stay or an increase in adjuvant chemotherapy completion is somewhat unexpected.

Some previous studies have suggested that VATS can facilitate the implementation of adjuvant chemotherapy, with a shorter time to adjuvant chemotherapy initiation or that patients would better tolerate chemotherapy [18-20]. However, only one study has found a significant increase in the chemotherapy completion rate in favor of VATS [20]. Other previous studies have found no differences in the completion of chemotherapy based on the surgical approach [21,22]. Interestingly, in the one study which reported the benefit of VATS on adjuvant chemotherapy completion, surgical complications were less frequent among patients who underwent VATS when compared to thoracotomy [20]. This observation further highlights a strong association between postoperative complications and adjuvant chemotherapy completion. Since the incidence of significant surgical complications in our study was similarly low in both groups, with only 6% of patients experiencing grade IIIb or IV complications, this may explain the absence of difference in the adjuvant chemotherapy completion between groups. Furthermore, in recent years, there has been an improvement in oncology supportive care including newer antiemetics, thus potentially enabling more patients to complete their adjuvant chemotherapy course.

To our knowledge, this is the first study to investigate the association between adjuvant chemotherapy completion and RATS. Some limitations will need to be factored in for generalizability. First, our institution performs a large volume of both thoracotomy and RATS. Early experiences with RATS have been marked by more surgical complications and bleeding than longstanding experiences with VATS [23]. Second, our analysis included pneumonectomy patients to represent a diverse patient population undergoing adjuvant chemotherapy; however, pneumonectomy is not generally feasible by RATS due to technical difficulty. Nevertheless, we have performed a propensity-matched analysis that addressed this issue. Finally, our study design does not capture patients who should have received adjuvant chemotherapy but did not receive it at all or did not receive it at our institution. However, the number of such patients is expected to be small.

## Conclusions

In conclusion, we found no difference in adjuvant chemotherapy completion between RATS patients and thoracotomy patients. However, the incidences of surgical complications in our study were low in both groups. While surgical technique did not significantly impact the chemotherapy completion, several factors including surgical complication, year of treatment, T-stage, and BMI were important predictors of chemotherapy completion. 
